# IL-21 Augments Rapamycin in Expansion of Alpha Fetoprotein Antigen Specific Stem-Cell-like Memory T Cells *in vitro*

**DOI:** 10.11604/pamj.2017.27.163.11072

**Published:** 2017-06-30

**Authors:** Victor Tunje Jeza, Xiaoyi Li, Jun Chen, Zhihui Liang, Adem Onago Aggrey, Xiongwen Wu

**Affiliations:** 1 Department of Immunology, Tongji Medical College, Huazhong University of Science and Technology, Wuhan, China; 2 Department of Medical Sciences, Technical University of Mombasa, Mombasa, Kenya; 3 Department of Mathematics and Physics, Technical University of Mombasa, Kenya

**Keywords:** Alpha Fetoprotein, Tscm cells, IL-21, Rapamycin, concurrent application, cancer immunotherapies

## Abstract

**Introduction:**

Alloreactive tumor specific T cells are important arsenals of the adaptive immune system in the fight against tumors. However, stem cell-like memory T cells (Tscm) provide the key to effective elimination of tumor cells. Methods for generating these T cell subsets already exist. However, they could be made more efficient. Further, they are expensive and unattainable to the resource poor laboratories. In this regard, we are hereby describing a novel in vitro allogeneic co-culture method for raising allo-restricted tumor specific Tscm cells that we developed.

**Methods:**

We started by obtaining PBLs that screened negative for HLA-A2 molecules from healthy donors followed by co-culture with T2/AFP cells to generate AFP peptide specific tumor-reactive T cells. Controls, IL-21 and/or rapamycin were applied to samples in 24 well plates. Samples were harvested and stained with anti-human CD3, CD8, CD44, CD62L, and HLA-A2/AFP dimer followed by flow cytometry analysis. Cell viability was measured by Trypan blue exclusion assay. One Way ANOVA and independent t test were used to compare the mean differences among and between groups where P values less than 0.05 were considered significant.

**Results:**

Our results show that rapamycin arrests the differentiation of, and expands AFP specific Tscm cells. Further, the expansion of Tscm cells is augmented in the presence of IL-21.

**Conclusion:**

IL-21 and Rapamycin can be used concurrently to raise and maintain antigen specific Tscm cells *in vitro* for purposes of augmenting immunotherapy strategies against cancers.

## Introduction

Cancer is a disease characterized by abnormal cell growth mediated by new protein molecule expression. Since our immune system has developed to eradicate foreign looking substances, it can protect us from cancer due to the abnormalities in the cells being seen as foreign. Cytotoxic T (Tc) cells are the major mediators of adaptive immunity against cancers. As such, they can be used in adaptive cellular therapy to eradicate tumors and are poised to be the most promising strategy where these Tc cells are taken from the cancer patient, expanded in vitro and transferred back to the patient [1]. The efficiency of this strategy is dependent on the stage of differentiation at which these cells are. At the effector stage, these cells can kill tumors cells. This stage is replenished by the earlier stages of differentiation which include central memory and stem cell-like memory Tc cells. In the presence of IL-2, the cells can differentiate quickly to get to the effector stage. However, the T cells in this case can become exhausted quickly and therefore become less efficient at eradicating tumors [2, 3]. Stem cell-like memory T cell subsets (Tscm) are early stage differentiated T cells that are antigen experienced subsets of T cells. They are characterized by expression of CD44^low^CD62L^high^ just like naïve T cells [4] but also express stem cell antigen-1 (Sca-1) and high levels of the antiapoptotic molecule B cell lymphoma 2 (Bcl-2), the βchain of the IL-2 and IL-15 receptor (IL-2Rβ), and the chemokine (C-X-C motif) receptor CXCR3 [5, 6]. Stemness attributes found in Tscm allows them to differentiate further [7] leading to generation of effector Tc cells steadily and continuously thereby giving them the opportunity to perpetually attack tumor cells [6, 8–12]. This increases the efficiency of these cells. It has been shown that treating Tc cells with IL-21 can confer stemness to Tc cells. Further, it has been suggested that treating or exposing these cells to small molecules that signal through Wnt [6, 13], Akt [1, 14–16] and mTOR inhibitors [12, 17–19] might contribute to the same effects. Other studies have shown that rapamycin can extend the lifespan of certain cells and organisms [20]. We therefore hypothesized that concurrent application of IL-21 and rapamycin-an mTOR inhibitor-may augment the stemness attributes of these T cell sub-sets. Our results show that IL-21 augments rapamycin in expanding and maintaining Tscm cells in vitro for long periods of time. In essence, we have developed a novel *in vitro*allogeneic co-culture method for raising allo-restricted tumor specific Tscm cells. This method is easy, inexpensive, straightforward and augmentative when compared to other methods that have been shown to also work such as the generation of long-lived antitumor CD8+ T cells using artificial antigen presenting cells [21], the use of IL-7 and IL-15 to generate Tscm cells [22] and the recently identified CD27-dependent pathway of T cell expansion [23].

## Methods

**HLA-A2 Blood Typing**: Samples of peripheral blood were obtained from healthy volunteers with informed consent and approval by the Ethical Committee of Tongji Medical College. One hundred micro-liters of each of the blood samples of interest were taken to new tubes and 1 μl of fluorescence isothiocyanate (FITC) conjugated BB7.2 antibody was added to each tube and mixed by pipetting. The samples were then incubated at 4°C for 40 minutes. Red blood cells (RBCs) were lysed using red blood cell lysis buffer and samples washed twice then resuspended in 300 μl phosphate buffered saline (PBS) followed by flow cytometry analysis. Only those blood samples that stained negative for BB7.2 were used in subsequent experiments.

**Preparation of HLA-A2/AFP dimer**: Constructed HLA-A2 dimer was expressed on dimer-721.221 cells as previously described [24] and the supernatant purified through protein A column. The purified dimer was labeled with FITC (Abcam-Japan) according to the manufacturer's instructions then loaded with AFP peptides at 4°C for 48 hours before use.

**Cell co-culture**: PBMCs were obtained from the HLA-A2 negative peripheral blood samples by density gradient centrifugation using Ficoll-Hypaque (density 1.077 g/ml) according to the method by McCoy [25]. The PBMCs were then washed twice in PBS, suspended in 10% FCS RPMI 1640 medium and allowed to adhere to flasks overnight at 37 ^°^C with 5% CO_2_ atmosphere so as to obtain the non-adherent cells which were then used as responder cells. T2 cells were pulsed with α-fetoprotein (AFP) peptides (Hangzhou Peptide Co. -China) for three hours to form T2/AFP followed by inactivation in Mitomycin C (30 μg/ml) and used as stimulator cells. The responder to stimulator ratio was 10:1 with responder cells (PBLs) being 2.5 x 10^6^ cells per well in 24 well plates. Rapamycin (CALBIOCHEM-USA) and IL-21 (PeproTech-USA) were applied singularly and concurrently while controls were left untreated with neither rapamycin nor IL-21. Samples were stained with APC/CY7 conjugated anti-human CD3 (E Bioscience-USA), PE/CY5 conjugated anti-human CD8 (Biolegend-USA), PE conjugated anti-human CD44 (E Bioscience-USA) and PE/CY7 conjugated anti-human CD62L (Biolegend-USA) and analyzed by flow cytometry. In another assay to analyze AFP-specific Tscm cells, samples were stained with APC/CY7 conjugated anti-human CD3, PE/CY5 conjugated anti-human CD8, PE conjugated anti-human CD44, PE/CY7 conjugated anti-human CD62L and FITC conjugated HLA-A2/AFP dimer and analyzed by flow cytometry.

**Trypan blue exclusion test for cell viability**: Trypan blue was kindly provided by Prof Li Dzuoya from the Department of Immunology. Staining and analysis were performed according to the methods by Coder DM and Johnson S [26–27]. Cell viability was calculated as follows: % viable cells = (number of viable cells/number of total cells) X 100.

**Statistical analysis**: One way ANOVA was used to determine the difference of the means among the controls and the IL-21 and/or rapamycin treated groups of the co-culture experiments for determining stemness maintenance over the study period. The independent t-test was used to determine the difference of the means between the control and IL-21 and/or rapamycin treated groups in the Trypan Blue exclusion test for cell viability. Values of P ≤0.05 were considered significant.

## Results

**Figure 1:**
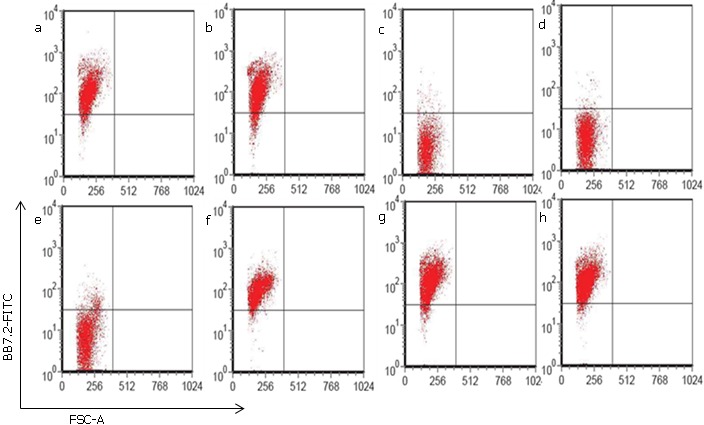
HLA-A2 Blood Typing. A 100 μl aliquot was taken from each of the samples and stained with the BB7.2 antibody. Flow cytometry was used to analyze the results. From these representative results of many, we can see that samples a, b, f, g, and h are positive for BB7.2 staining meaning that the PBMCs in these samples express the HLA-A2 molecules. In contrast, samples c, d, and e are negative for the BB7.2 staining and therefore show that these particular samples do not have the HLA-A2 molecules expressed on the PBMC surfaces.

**Figure 2:**
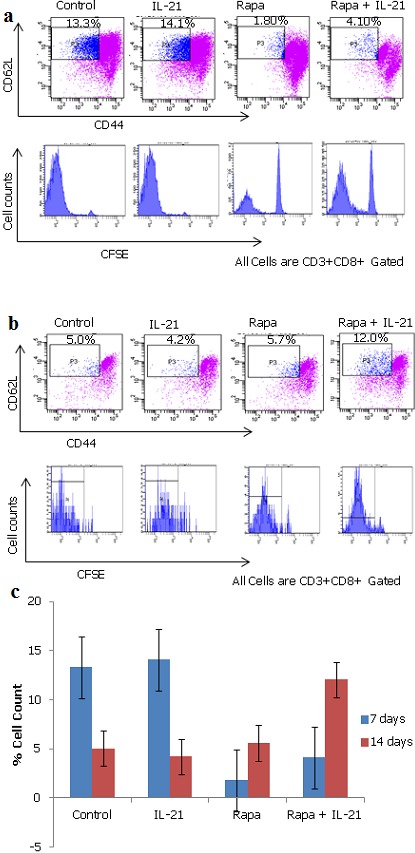
IL-21 augments rapamycin (Rapa) in induction of Tscm cells (no antigen specificity here) with increased proliferation. After 7 and 14 days of Co-culture (Figure 2a and 2b (top two panels and bottom two panels respectively)), samples were harvested and analyzed by flow cytometry. Since there is no specificity, the initial Tscm cells are seen to be very many in the control group and IL-21 treated groups. They are then seen to drop significantly in those two groups (Table 1 for statistics) while they were maintained for longer periods and increased in number (proliferated) in the presence of rapamycin. This proliferation is seen to be augmented when rapamycin is combined with IL-21 (Figure 2c (bar graph)).

**IL-21 augments rapamycin in induction of non-specific Tscm cells with initial increased proliferation**: To determine the consequences of singular and concurrent application of IL-21 and rapamycin on Tscm cells, HLA-A2 negative PBLs (Figure 1) were stained with CFSE and co-cultured with T2/AFP cells for a period of two weeks. Samples were harvested on day 7 and day 14 and stained with APC/CY7 conjugated anti-human CD3, PE/CY5 conjugated anti-human CD8, PE conjugated anti-human CD44, and PE/CY7 conjugated anti-human CD62L and analyzed by flow cytometry. Results show that rapamycin increases the non-specific (non-specific since expression of CD44^low^CD62L^high^ molecules could be found in either naïve or antigen experienced T cells) Tscm cells and this increase is augmented in the presence of both rapamycin and IL-21 (Figure 2). The mean difference in time were statistically significant (P= 0.0004, 3.18 X 10-6, 7.71 X 10-7 and 0.0001 for control, IL-21, rapamycin and IL-21 + rapamycin treated groups respectively). The mean difference between control and IL-21 treated groups was not statistically different while those between control and the other groups were statistically significant (Table 1).

**Table 1:** Statistical analysis of the mean difference between treated groups for non-specific Tscm cell experiments Multiple Comparisons Dependent Variable: values LSD

(I) factor1	(J) factor1	Mean Difference (I-J)	Std. Error	Sig.	95% Confidence Interval	
					Lower Bound	Upper Bound
**1(control)**	2	0.0000	0.07658	1.000	-0.1623	0.1623
	3	5.4650*	0.07658	0.000	5.3027	5.6273
	4	1.1000*	0.07658	0.000	0.9377	1.2623
**2(IL-21)**	1	0.0000	0.07658	1.000	-0.1623	0.1623
	3	5.4650*	0.07658	0.000	5.3027	5.6273
	4	1.1000*	0.07658	0.000	0.9377	1.2623
**3(rapa)**	1	-5.4650*	0.07658	0.000	-5.6273	-5.3027
	2	-5.4650*	0.07658	0.000	-5.6273	-5.3027
	4	-4.3650*	0.07658	0.000	-4.5273	-4.2027
**4(IL-21 +rapa)**	1	-1.1000*	0.07658	0.000	-1.2623	-0.9377
	2	-1.1000*	0.07658	0.000	-1.2623	-0.9377
	3	4.3650*	0.07658	0.000	4.2027	4.5273

Based on observed means The error term is Mean Square (Error) = 0.018

* The mean difference is significant at the 0.05 level

**Figure 3:**
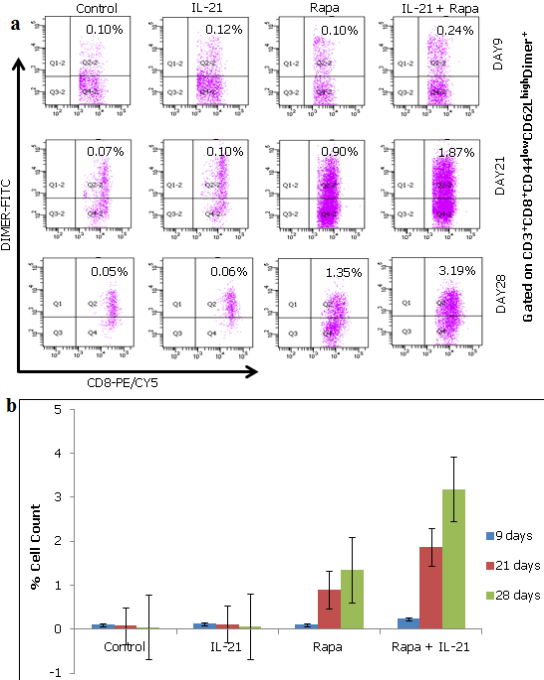
IL-21 augments rapamycin (Rapa) in maintaining and expanding of AFP antigen specific Tscm cells. After 9, 21, and 28 days of co-culture (Figure 3a (top, middle, and bottom panels respectively)), samples were harvested and analyzed by flow cytometry. HLA-A2 dimer (DIMER-FITC) was used to pick up AFP antigen specific Tscm cells. AFP antigen specific Tscm cells are maintained and increased significantly over longer periods in the presence of rapamycin which is augmented when rapamycin is combined with IL-21 (Figure 3b (bar graph)).

**IL-21 augments rapamycin in maintaining and expanding of AFP antigen specific Tscm cells**: We next sought to analyze the consequences of singular and concurrent application of IL-21 and rapamycin on antigen experienced AFP-Specific Tscm cells. To do this, HLA-A2 negative PBLs (Figure 1) (without staining with CFSE) were co-cultured with T2/AFP cells for a period of four weeks with the application of IL-21 and/or rapamycin. The control group had neither IL-21 nor rapamycin applied. Samples were harvested on days 9, 21, and 28 and stained with APC/CY7 conjugated anti-human CD3, PE/CY5 conjugated anti-human CD8, PE conjugated anti-human CD44, PE/CY7 conjugated anti-human CD62L, and FITC conjugated HLA-A2/AFP dimer and analyzed by flow cytometry. The aim of using HLA-A2/AFP dimer here is to be able to pick up and analyze antigen experienced T cells only. Our results show that rapamycin maintains and expands AFP antigen specific Tscm cells. This maintenance and expansion is augmented in the presence of IL-21 (Figure 3). We found that the mean difference between treatment groups and time were statistically significant (Table 2, Table 3).

**Table 2:** Statistical analysis of the mean difference between treated groups for AFP antigen specific Tscm cell experiments

(I) factor1	(J) factor1	Mean Difference (I-J)	Std. Error	Sig.	95% Confidence Interval	
					Lower Bound	Upper Bound
**1(control)**	2	-0.0244*	0.00653	0.001	-0.0379	-0.0110
	3	-0.7089*	0.00653	0.000	-0.7224	-0.6954
	4	-1.6922*	0.00653	0.000	-1.7057	-1.6788
**2(IL-21)**	1	0.0244*	0.00653	0.001	0.0110	0.0379
	3	-0.6844*	0.00653	0.000	-0.6979	-0.6710
	4	-1.6678*	0.00653	0.000	-1.6812	-1.6543
**3(rapa)**	1	0.7089*	0.00653	0.000	0.6954	0.7224
	2	0.6844*	0.00653	0.000	0.6710	0.6979
	4	-0.9833*	0.00653	0.000	-0.9968	-0.9699
**4(IL-21 + rapa)**	1	1.6922*	0.00653	0.000	1.6788	1.7057
	2	1.6678*	0.00653	0.000	1.6543	1.6812
	3	0.9833*	0.00653	0.000	0.9699	0.9968

Based on observed means The error term is Mean Square (Error) = 0.000

* The mean difference is significant at the 0.05 level

Dependent Variable: values LSD

**Table 3:** Statistical analysis of the mean difference in time between treated groups for the AFP antigen specific Tscm cell experiments Multiple Comparisons Dependent Variable: values LSD

(I) factor2	(J) factor 2	Mean Difference (I-J)	Std. Error	Sig.	95% Confidence Interval	
					Lower Bound	Upper Bound
**1(9days)**	2	-0.5983*	0.00565	0.000	-0.6100	-0.5867
	3	-1.0217*	0.00565	0.000	-1.0333	-1.0100
**2 (21days)**	1	0.5983*	0.00565	0.000	0.5867	0.6100
	3	-0.4233*	0.00565	0.000	-0.4350	-0.4117
**3(28days)**	1	1.0217*	0.00565	0.000	1.0100	1.0333
	2	0.4233*	0.00565	0.000	0.4117	0.4350

Based on observed means The error term is Mean Square (Error) = 0.000

* The mean difference is significant at the 0.05 level

**Figure 4:**
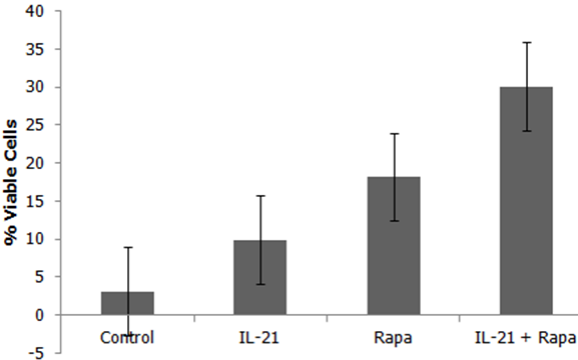
IL-21 augments rapamycin in maintaining viability of AFP antigen specific Tscm cells. After 28 Days of Co-culture, samples were harvested and stained with 0.4% Trypan Blue. Rapamycin increased the viability of AFP antigen specific Tscm cells which was augmented in the concurrent treatment of IL-21 and rapamycin. The difference between viable cells in control compared with IL-21, Rapa, and IL-21 + Rapa were statistically significant (P = 0.0003, 1.98X10-5, 1.53X10-6 respectively).

**IL-21 augments rapamycin in maintaining viability of AFP antigen specific Tscm cells**: Samples were harvested on day 28 and washed in PBS twice. They were then stained with Trypan Blue and incubated at room temperature for 5 minutes. Cell viability was determined in each treated group and the results show that application of IL-21 and/or rapamycin maintains cell viability compared to the control group showing a statistical significant difference (p=0.0003, 1.98X10-5, 1.53X10-6 respectively). The maintenance of cell viability was more superior in concurrent application of IL-21 and rapamycin (Figure 4).

## Discussion

Previous research has shown that the human T cell repertoire can recognize an AFP peptide as a HLA-A2.1 restricted epitope and is expressed in certain tumors such as hepatocelullar carcinoma and germ cell tumors [28, 29]. Therefore, we employed the AFP peptides to generate HLA-A2 allo-restricted tumor antigen specific T cells. To do this, T2 cells were pulsed with the AFP peptides for three hours at 37°C with 5% CO2 atmosphere containing incubator to generate T2/AFP cells which were then co-cultured with PBLs that were negative for expression of HLA-A2 molecules (Figure 1). From here, we assessed the ability, either singularly or concurrently, of the biological and pharmacological agents IL-21 and rapamycin, respectively, to arrest the differentiation of these AFP antigen specific activated cells so as to sparingly reach the effector cell stage and largely retain as well as expand the Tscm subset. Our preliminary experiments looked at the consequences of IL-21 and rapamycin, as applied either singularly or concurrently, to arrest T cell differentiation at the Tscm stage. After two weeks of co-culture, we found that rapamycin had relatively higher Tscm subset cells compared to IL-21 alone. The magnitude of change in the cell numbers was even more in the concurrent application of IL-21 and rapamycin group (Figure 2). We found that at the end of week one, there were more Tscm cell numbers in the control group and the IL-21 treated group. This is the case since these cells had no specificity. As time progressed and non-antigen specific T cells (i.e. naïve T cells) died out, the situation changed (Figure 2 B (bottom two panels)). We reasoned that there might be non-specific Tscm cells being included in the analysis due to the absence of specificity in the system. To seek for answers, we repeated the experiment and incorporated staining with the antigen specific dimers -HLA-A2/AFP dimers- in conjunction with the other antibodies i.e. anti-human CD3, anti-human CD8, anti-human CD44, and anti-human CD62L staining. Here, the HLA-A2/AFP dimers provide a mechanism for picking up and analyzing AFP antigen experienced T cells only. In this case, we found that the trends from the analysis were either a continuous increase or decrease of the Tscm cells in the different experimental groups (Figure 3). AFP-specific Tscm cells increased significantly from a low of 0.10% on day 9 to 1.35% by day 28 in the presence of rapamycin. The expansion of these cells was more than two fold in the concurrent application of IL-21 and rapamycin under the same period. In the control and IL-21 treated groups, the number of cells decreased over time continuously (Figure 3 bar graph). Here, the results show that IL-21 augments rapamycin in expanding AFP antigen specific Tscm cells. Taken together, these results show that rapamycin quickly destroys naïve T cells but maintains antigen experienced T cells at the early differentiation stage for longer periods thereby maintaining their stemness and allows them to proliferate at the same time. This proliferation is enhanced in the presence of both IL-21 and rapamycin. The mechanism that allows this to happen remains to be elucidated.

We further wanted to know the viability of the cells being maintained by IL-21 and rapamycin. To do this, we employed the Trypan blue exclusion assay. Cells were harvested on day 28 and washed in PBS twice then stained with Trypan Blue and incubated at room temperature for five minutes. Cell viability was determined in each treated and control groups. The results show that the viability of the cells in co-culture was enhanced by the addition of rapamycin while, in comparison, it was augmented by addition of IL-21 and rapamycin concurrently (Figure 4). This might be explained by the fact that rapamycin regulates fatty acid metabolism in activated T cells thereby regulating their proliferation [30, 31] which leads to a much slower exhaustion process. In application of rapamycin for induction of Tscm cells, dosage seems to be paramount as demonstrated in our results (data not shown). This phenomenon is also supported by work from other laboratories such as that of Benjamin and colleagues who showed that the incomplete inhibition on mTORC1 is a dose dependent phenomenon that could have devastating effects in cancer patients [32]. Further support of our results comes from others who have shown that the effect of mTOR inhibition depends on the dose range and kinetics of the treatment. Administration of a very high dose of rapamycin prevented CD8+ T cell expansion whereas the duration and time points of rapamycin treatment impact the quantity and quality of memory T cell responses [17, 33]. In addition, optimal doses of rapamycin were seen to increase the life span of middle aged mice [20]. Some studies suggest that blocking mTOR not only mediates immunosuppression in transplant rejections and autoimmune disorders, but also boosts immunity under selective conditions and imparts other aspects of T cell homeostasis and functions [18]. Further, it has been reported that suppression of the mTOR pathway, an established nutrient sensor, combined with activation of canonical Wnt-β-catenin signaling, allows for the ex vivo maintenance of human and mouse long-term HSCs under cytokine-free conditions. The same group also showed that the combination of two clinically approved medications that together activate Wnt-β-catenin and inhibit mTOR signaling increases the number (but not the proportion) of long-term HSCs in vivo [34]. Here, our results have shown that IL-21 and the mTOR inhibitor rapamycin combined together increase both the number and proportion of Tscm cells by the fourth week of co-culture (Figure 3). In contrast, it has been shown that in vivo, mTOR inhibition promotes T cell anergy under conditions that would normally induce active priming, indicating that mTOR has a central role in dictating the decision between T cell activation and anergy [35, 36]. Could it be that these observations were made under the influence of high doses of rapamycin application? The emergence of memory and effector CD8+ T cells is independent of, but functional quality shaped by, IL-21 [37–39]. Yi et al [37] have also argued that although the direct effects of IL-21 on CD8+ T cells may shape these responses, IL-21 imparts many immunologic functions and therefore the indirect actions of IL-21 on other cells of the immune system may also influence the outcome of the CD8+ T cell response. We found that IL-21 alone has a reduced ability to retain AFP-specific Tscm cells when compared to rapamycin alone or its concurrent application with rapamycin (Figure 3).

## Conclusion

Rapamycin application expands AFP-specific Tscm cells after AFP antigen specific stimulation of naïve T cells and maintains viability of co-cultured cells better than IL-21. In addition, IL-21 augments rapamycin in the expansion of AFP-specific Tscm cells and therefore these two agents may have an increased potential for tumor immunotherapy when applied concurrently. Essentially, this work provides a new co-culture method by which antigen specific Tscm cells can be raised and their stemness attributes maintained

### What is known about this topic

- It is know that IL-21 help in generating and maintaining Tscm cells;
- It is known that Tscm cells are better eradicators of cancers and have a great potential for clinical use against cancers.

### What this study adds

- This study provides a novel method for generating, expanding and maintaining antigen specific Tscm cells;
- It contributes towards the search for immunotherapy against cancers.

## Competing interests

The authors declare no competing interest.
